# Inference of Admixture Origins in Indigenous African Cattle

**DOI:** 10.1093/molbev/msad257

**Published:** 2023-11-23

**Authors:** Kwondo Kim, Donghee Kim, Olivier Hanotte, Charles Lee, Heebal Kim, Choongwon Jeong

**Affiliations:** The Jackson Laboratory for Genomic Medicine, Farmington, CT, USA; Department of Agricultural Biotechnology and Research Institute of Agriculture and Life Sciences, Seoul National University, Seoul, Republic of Korea; School of Biological Sciences, Seoul National University, Seoul, Republic of Korea; LiveGene, International Livestock Research Institute (ILRI), Addis Ababa, Ethiopia; The Centre for Tropical Livestock Genetics and Health (CTLGH), The Roslin Institute, The University of Edinburgh, Midlothian, UK; School of Life Sciences, University of Nottingham, Nottingham, UK; The Jackson Laboratory for Genomic Medicine, Farmington, CT, USA; Department of Agricultural Biotechnology and Research Institute of Agriculture and Life Sciences, Seoul National University, Seoul, Republic of Korea; Interdisciplinary Program in Bioinformatics, Seoul National University, Seoul, Republic of Korea; eGnome, Inc., Seoul, Republic of Korea; School of Biological Sciences, Seoul National University, Seoul, Republic of Korea

**Keywords:** African cattle, genome, admixture, introgression

## Abstract

Present-day African cattle retain a unique genetic profile composed of a mixture of the *Bos taurus* and *Bos indicus* populations introduced into the continent at different time periods. However, details of the admixture history and the exact origins of the source populations remain obscure. Here, we infer the source of admixture in the earliest domestic cattle in Africa, African taurine. We detect a significant contribution (up to ∼20%) from a basal taurine lineage, which might represent the now-extinct African aurochs. In addition, we show that the indicine ancestry of African cattle, although most closely related to so-far sampled North Indian indicine breeds, has a small amount of additional genetic affinity to Southeast Asian indicine breeds. Our findings support the hypothesis of aurochs introgression into African taurine and generate a novel hypothesis that the origin of indicine ancestry in Africa might be different indicine populations than the ones found in North India today.

## Introduction

African cattle are an essential component of the traditional herder economies in many regions of Africa. Archaeological records document the earliest cattle introduction into North Africa around 8,000 yr before the present (yBP) from the Levant, its spread South of the Sahara to West and East Africa around 4,500 yBP, and finally to South Africa around 2,000 yBP (Smith 2021). The earliest cattle on the continent are considered to be taurine (*Bos taurus*), which originated from the domestication of aurochs, *Bos primigenius primigenius* in the Near East ∼10,000 yBP (Bollongino et al. 2012). Following this initial spread of the taurine cattle, a later introduction of and an admixture with the indicine cattle (*Bos indicus* or zebu) shaped the contemporary genetic diversity of African cattle breeds. The indicine cattle first appeared in South Asia ∼8,000 yBP, ca. 2 millennia later than the taurine cattle in the Near East, and are assumed to have originated at least partially from *B. p. nomadicus*, a South Asian aurochs subspecies (Utsunomiya et al. 2019). Taurine and indicine cattle species are phenotypically distinct and substantially diverged from each other, with divergence time estimates ranging between 200,000 and 1,000,000 yr reflecting their descent from distinct aurochs populations (Loftus et al. 1994; MacHugh et al. 1997; Achilli et al. 2008; Bibi 2013).

The genetic origin of the African taurine cattle remains in debate, especially with regard to why the present-day African taurine breeds are genetically differentiated from the Eurasian taurine ones to a substantial degree. Three main hypotheses have been proposed to explain the origins of African taurine cattle: (i) the African taurine cattle are descents from a domesticated Eurasian taurine population in the Near East and underwent significant genetic drift and natural selection over time (Mwai et al. 2015), (ii) it was independently domesticated from an aurochs population in North Africa (Bradley et al. 1996; Wendorf and Schild 1998; Troy et al. 2001), and (iii) it descended from an admixture between the Near Eastern taurine cattle and local African aurochs (*B. p. africanus*) (Decker et al. 2014; Brass 2018; Pitt et al. 2019). Previous genetic studies overall supported a single domestication event within the taurine cattle, rejecting the independent domestication of the African taurine cattle. Indeed, the African taurine cattle show a limited amount of maternal genetic diversity, represented by their mitochondrial DNA, embedded within the much higher diversity among present-day Near Eastern cattle (Troy et al. 2001; Bonfiglio et al. 2012). Also, an inference based on genome-wide genotype data of worldwide cattle breeds rather supports a single taurine domestication scenario over 2 events within the taurine cattle (Pitt et al. 2019).

More recent genetic studies have reported suggestive evidence for an admixture with a local aurochs population. A population graph of worldwide cattle breeds based on ∼43,000 single-nucleotide polymorphisms (SNPs) inferred an admixture from a deep taurine branch to a common ancestor of several African cattle breeds and interpreted this signal as an African aurochs introgression. However, the inferred population graph did not explicitly take into account the indicine ancestry of the African cattle and relied only on a low-density SNP panel (Decker et al. 2014). More recently, an analysis of ancient cattle genomes from Europe and the Near East showed the affinity between North African aurochs (Moroccan aurochs, dated to ∼9,000 yBP) and Neolithic, Bronze, and Iron age cattle without indicine introgression in the Southern Levant, a geographic area close to the entry point of taurine cattle to the African continent, suggesting an aurochs admixture into ancient Levantine cattle (Verdugo et al. 2019). However, it remains unexplored whether this admixture was also shared by the ancestors of the African taurine cattle and/or whether additional local aurochs introgression might have occurred in Africa.

The admixture between indicine and taurine cattle is a key feature to explain the adaptive diversity of the African cattle, with at least 150 breeds recognized with diverse phenotypes (Mwai et al. 2015). However, so far only a few studies have addressed the details of taurine × indicine admixture event(s) at the whole genome level. Our previous study showed its impacts on the adaptation of African cattle, while suggesting that the extensive crossing with local taurine occurred at least 2 to 3 centuries (Kim et al. 2020) after the main introduction of the indicine cattle started around 700 AD in East Africa (Epstein 1971; Gifford-Gonzalez and Hanotte 2011). In the West African population, the timing of indicine admixture was estimated to ∼500 yr ago using genome-wide SNP genotype data (Flori et al. 2014). These studies use indicine populations found in today's North India as a proxy to the population that have contributed to the taurine × indicine admixture in Africa, assuming that indicine ancestry in Africa originated from the ancestral population of present-day North Indian indicine. However, the assumption has not been fully explored and the exact source of the indicine ancestry in Africa remains speculative.

Here, we analyzed published data consisting of 595 modern and 38 ancient genomes including cattle, aurochs, and other bovid species across the world from publicly available resources. Using this data set, we investigate the genetic profiles of the taurine and indicine ancestries of the African cattle in fine resolution. By doing so, we test the hypothesis of “local aurochs introgression” in today's African cattle, estimate its admixture proportion in African taurine populations, and characterize the indicine ancestry of the African cattle in comparison with the indicine cattle around the world.

## Results

### The Genetic Profile of African Cattle Populations

**Fig. 1.**
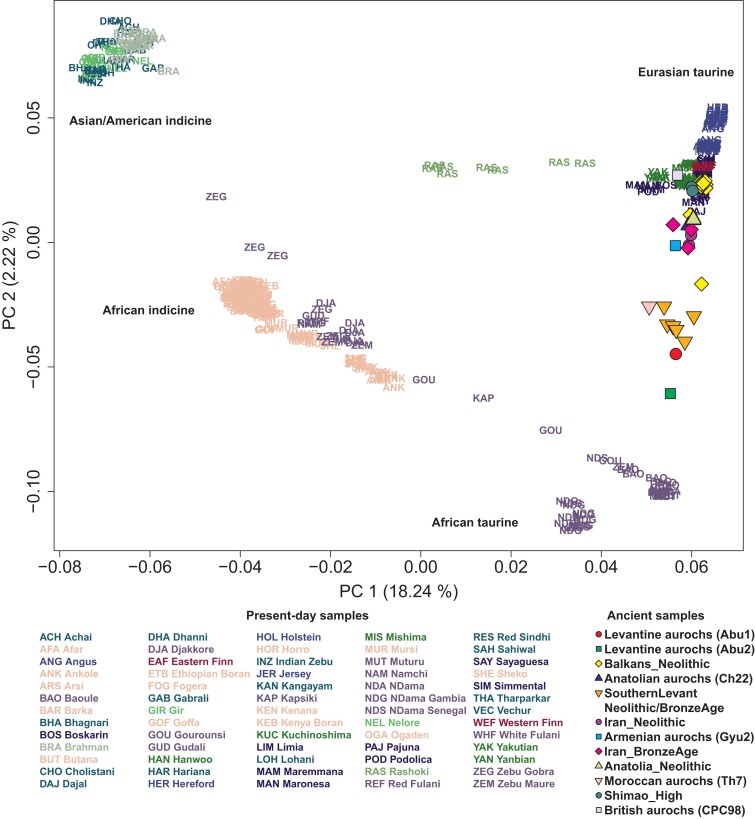
PCA of worldwide cattle breeds excluding Chinese ones. We calculated PCs from 394 present-day cattle individuals, including both taurine and indicine cattle but excluding Chinese breeds for a clearer separation within the taurine cattle. Each present-day cattle individual is marked by a 3-letter code representing its breed name. Ancient cattle and aurochs individuals are projected onto the top 2 PCs and marked by color-filled symbols. The proportion of variance explained by each PC is indicated in the axis labels.

For this study, we compiled publicly available whole genome sequences of 595 present-day Bovini individuals (543 cattle and 52 outgroup individuals) and 38 ancient cattle and aurochs individuals, resulting in 5,581,829 high-quality autosomal transversion SNPs (supplementary table S1, Supplementary Material online; see Materials and Methods). To explore the genetic profiles of African cattle breeds, we first calculated the principal components (PCs) of present-day worldwide cattle breeds and projected ancient cattle and aurochs from published studies (Park et al. 2015; Chen et al. 2018; Verdugo et al. 2019) to the calculated PCs. African populations are distributed along the first PC (PC1) (Fig. 1), the main axis of variation that separates Eurasian taurine and Asian/American indicine populations. Ancient southern Levantine individuals fall closer along PC2 to African taurine populations than Eurasian taurine ones. Among the 6 aurochs individuals, Moroccan (Th7 from the Taghit Haddouch site in Morocco) and Levantine aurochs (Abu1 and Abu2 from the Abu Gosh site in Israel) are more closely located to African taurine clusters than the other aurochs: Armenian (Gyu2 from the Gyumri site in Armenia), Anatolian (Ch22 from the Çatalhöyük site in Türkiye), and British aurochs (CPC98 the Carsington Pasture Cave site in England). The genetic cline formed by African indicine and taurine populations, when being extrapolated, does not seem to point to the cluster of Asian/American indicine (Fig. 1; supplementary fig. S1, Supplementary Material online).

### Evidence of a Local Aurochs Admixture in African Taurine Cattle

To test the hypothesis that a local aurochs population significantly contributed to the African taurine populations, we first selected African taurine breeds without a recognizable level of indicine admixture based on *f*_4_-statistics of the form *f*_4_(African buffalo, Sahiwal; African cattle, Eurasian Taurine). Only Muturu (*n* = 9) and N’Dama (*n* = 1), among the 28 African cattle breeds in our data set, show no extra genetic affinity with an indicine breed Sahiwal against the 4 ancient Eurasian taurine populations (Anatolia_Neolithic, Balkans_Neolithic, Iran_BronzeAge, and Shimao_High) (supplementary table S1, Supplementary Material online) as well as the present-day Simmental breed, thus showing no evidence of an indicine admixture (*Z* > −3 for all 5 taurine groups) (supplementary table S2, Supplementary Material online). Based on these results, we chose Muturu and N’Dama as proxies of the African taurine before the indicine admixture. We then examined the genetic affinity of Muturu, N’Dama, and Eurasian taurine breeds to the 4 aurochs (Anatolian, Armenian, British, and Moroccan aurochs; Levantine aurochs were excluded due to their low coverage) using outgroup *f*_3_- and *f*_4_-statistics. The Moroccan aurochs show the highest genetic affinity with Muturu and N’Dama, while the European aurochs show the highest genetic affinity with present-day European breeds such as Hereford and Angus (supplementary figs. S2 and S3 and tables S3 and S4, Supplementary Material online).

**Fig. 2.**
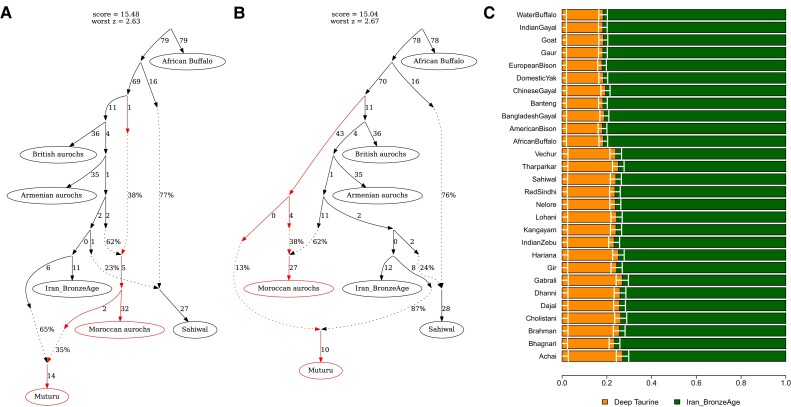
Admixture modeling of Muturu. We present 2 best population graphs, obtained from qpGraph, involving a gene flow into Muturu and adequately fitting the observed data. A) Topology B-2 models Muturu as a mixture of Iran_BronzeAge and the Moroccan aurochs, the latter of which received a gene flow from a deep taurine branch. B) Topology B-3 models Muturu as a mixture of Iran_BronzeAge and a deep taurine branch partially shared with the Moroccan aurochs. C) qpAdm-based estimates of the deep taurine admixture proportion in Muturu. We modeled Muturu as a mixture of Iran_BronzeAge and either an indicine or an outgroup Bovini species (marked on the left side) utilizing the fact that they can serve as a proxy for the unsampled deep taurine branch if none of the reference populations were more deeply branching than the deep taurine in Muturu (see Materials and Methods). Horizontal bars represent ±1 standard error measures estimated by 5-cM block jackknifing.

To test and quantify the potential aurochs introgression in the African taurine cattle, we investigated population graphs that could explain the relationship of the following 7 populations using qpGraph (Patterson et al. 2012): African buffalo, Sahiwal, Armenian aurochs, British aurochs, Moroccan aurochs, Iran_BronzeAge, and Muturu. In the backbone graph without Muturu, the Moroccan aurochs derives about a third of its ancestry from a deep taurine branch splitting earlier than the split between the British aurochs and the others (topology A-1; supplementary fig. S4, Supplementary Material online). The best graph for Muturu without involving a gene flow from a deep taurine lineage cannot adequately explain the observed relationship between populations (topology B-1; supplementary fig. S5, Supplementary Material online). The graph model becomes significantly improved and adequately fits the observed data by adding a gene flow into Muturu from branches leading to the Moroccan aurochs (empirical *P* = 0.001 for a significant improvement of the model fit in both topologies B-2 and B-3 compared with B-1) (Fig. 2A and B). The contribution from the deep taurine branch, either indirectly via the Moroccan aurochs (B-2) or directly (B-3), is estimated to be ∼13%. We obtained qualitatively same results when replacing Iran_BronzeAge with Anatolia_Neolithic (supplementary fig. S6, Supplementary Material online).

To quantify the robustness of the Moroccan aurochs-related introgression signal, we simulated 100 sets of genome-wide genotype data of the 7 populations used in the qpGraph for each of the best topologies without (B-1) and with (B-2 and B-3) a gene flow into Muturu. For the data sets simulated without a gene flow (based on the B-1 topology), we counted the number of simulated data sets for which graphs with a gene flow (B-2 or B-3) show a significantly better fit than the true graph B-1 as a measure of false positive for the introgression signal. For the data sets simulated with a gene flow, we counted the number of simulated data sets for which graphs without a gene flow (B-1) explain data without a significant reduction in the model fit as a measure of false negative. We find no simulation leading to the false-positive discovery of introgression, supporting the robustness of the introgression signal we report (Table 1). The false-negative rates are also low for both topologies: 0% (0/100) for B-2 and 1% (1/100) for B-3 (Table 1). The results remain qualitatively unchanged when using a 10-fold greater split time between African buffalo and cattle, supporting the robustness of our simulation with regard to the modeling of the outgroup species (Table 1).

**Table 1:** Robustness assessment of a deep taurine-related introgression signal into Muturu using simulated genotype data

True graph	B-1		B-2	B-3
Category	False positive (compared with B-2)	False positive (compared with B-3)	False negative	False negative
Rate (split at 300 kya)	0% (0/100)	0% (0/100)	0% (0/100)	1% (1/100)
Rate (split at 3 Mya)	0% (0/100)	0% (0/100)	0% (0/100)	0% (0/100)

To test the robustness of the identified introgression signal from a deep taurine into Muturu, we simulated 100 sets of genotype data for each of the 3 competing topologies: 1 without any gene flow into Muturu (B-1) and 2 with gene flows (B-2 and B-3). In each topology, we incorporated 2 distinct split times between the African buffalo and cattle, specifically 300 thousand years ago (kya) and 3 million years ago (Mya). For each simulated data set, we evaluated model fit for all 3 topologies. False-positive measurements were determined by counting the number of simulated data sets where graphs with gene flows (B-2 or B-3) significantly (*P* < 0.05) outperformed the true graph (B-1) in explaining the data. False-negative rates were calculated as the proportion of simulated data sets for which the graph without a gene flow (B-1) adequately explained the data without a significant reduction in the model fit from the true graph (*P* > 0.05).

We also applied closely related but different methods to replicate our qpGraph-based findings on the aurochs introgression signal in the African taurine. First, the qpAdm modeling of Muturu as a mixture of Iran_BronzeAge and the Moroccan aurochs, corresponding to the topology B-2, adequately fits Muturu with the Moroccan aurochs contribution comparable with the qpGraph-based estimate (qpAdm *P* = 0.076 with 38.2 ± 2.5% contribution from the Moroccan aurochs; 35% from qpGraph). Second, we applied a qpAdm-based method for quantifying the amount of a deep lineage introgression without a sample on that lineage (Skoglund et al. 2017). We obtain an 18% to 19% contribution using outgroups more distant to the taurine-indicine split (e.g. African buffalo), slightly higher than but largely comparable with the qpGraph-based estimate (Fig. 2C; supplementary table S5, Supplementary Material online). Using South Asian indicine breeds, we obtain 24% to 27% contribution, which is in fact comparable with the above estimate considering that the South Asian indicines derive about a quarter of their ancestry from the taurine branch (Fig. 2A and B; supplementary fig. S5 and table S5, Supplementary Material online). Third, an automated population graph search using TreeMix (Pickrell and Pritchard 2012) also infers a gene flow from the Moroccan aurochs into Muturu with 40% proportion comparable with 35% from qpGraph (B-2 topology) (supplementary fig. S7, Supplementary Material online).

### Sources of the Indicine Admixture in African Cattle

**Fig. 3.**
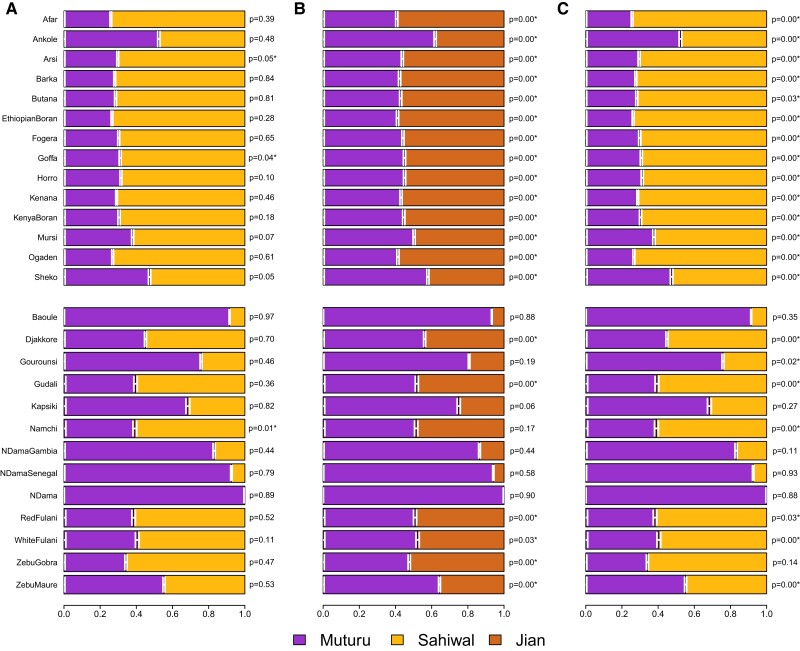
Admixture modeling of admixed African cattle breeds with qpAdm. We performed 2-way admixture modeling using qpAdm for African cattle breeds, with Muturu as the African taurine source and either North Indian indicine Sahiwal (A, Model I and C, Model III) or Southeast Asian indicine Jian (B, Model II) as the indicine source. For A) and B), we used the base set of reference populations: African buffalo, British aurochs, Balkan_Neolithic, and Shimao_High. For C), we add Jian as an additional reference population to the base set. Upper and lower panels harbor East and West African breeds, respectively. In each panel, the target African cattle breed is marked on the left side and the qpAdm *P*-value is indicated on the right side. *P*-values with asterisk (*) refer to unsuccessful models (*P* < 0.05). Horizontal bars represent ±1 standard error measures estimated by 5-cM block jackknifing.

To characterize the genetic profile of the indicine ancestry in African cattle in a fine resolution, we sequentially performed multiple qpAdm tests with diverse sets of source/target (left) and reference (right) populations for each African cattle breed (see Materials and Methods). As a base reference population set, we heuristically chose 4 groups: African buffalo as a distant outgroup to both taurine and indicine lineages, British aurochs, Balkans_Neolithic and Shimao_High to tag the taurine lineage against the indicine one as well as to distinguish Eurasian and African taurines. Using the base reference set, we tested if 2-way admixture models of Muturu + Indicine could explain various African cattle breeds when using South Asian indicine breed Sahiwal (Model I) or Southeast Asian indicine breed Jian (Model II) as an indicine source. Model I (Muturu + Sahiwal) adequately fits African cattle breeds (*P* > 0.05) in 24 out of 27 populations except Arsi, Goffa, and Namchi (Fig. 3A; supplementary table S6, Supplementary Material online). In sharp contrast, Model II using a divergent Southeast Asian indicine breed (Jian) as a source fails to explain most African cattle breeds (20 out of 27) excluding a few with a low admixture proportion of indicine (Baoule, Gourounsi, Kapsiki, Namchi, N’Dama Gambia, N’Dama Senegal, and N’Dama) (Fig. 3B; supplementary table S6, Supplementary Material online). These results confirm that the true indicine source of the African cattle is closely related to present-day indicine breeds from North India while not matching to divergent Southeast Asian indicines. In addition, they corroborate our qpGraph analysis in which Southeast Asian indicines do not form a simple sister clade with South Asian indicines (supplementary fig. S8, Supplementary Material online).

We then repeated our Muturu + Sahiwal admixture modeling after adding Jian as an additional reference to the base set (Model III). Interestingly, Model III failed to fit the genetic profile of the African cattle breeds in most cases (21 out of 27 breeds with *P* < 0.05), although the same source combination (Muturu + Sahiwal) was sufficient to model most of African breeds in Model I (Fig. 3C; supplementary table S6, Supplementary Material online). We find that Model III fails because the target African cattle have affinity with Jian compared with the proposed model of Muturu + Sahiwal. We observe consistent results when replacing Jian in the reference set with any other southern Chinese cattle breeds with a high level of indicine ancestry (Chen et al. 2018) (supplementary table S7, Supplementary Material online). In agreement with the allele–frequency-based qpAdm results, we also observed a small amount of Southeast Asian indicine contribution in most African breeds (0.8% to 6.5%), using a haplotype-sharing-based method GLOBETROTTER (supplementary fig. S9 and table S8, Supplementary Material online).

**Fig. 4.**
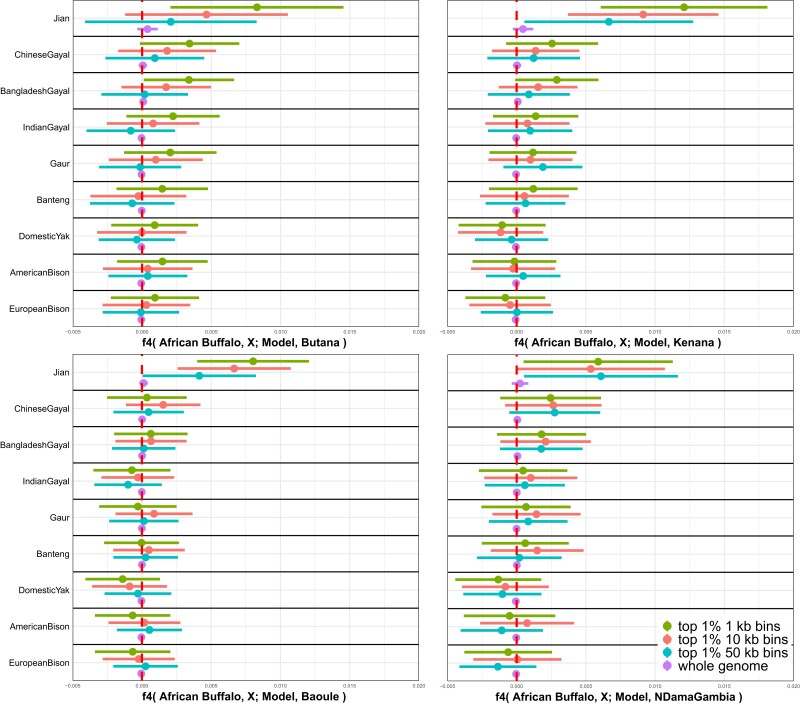
Enrichment of outgroup affinity in African cattle genomic segments divergent from the simple admixture model. We identified genomic segments in African cattle divergent from Model I (Muturu + Sahiwal) by taking top 1% genomic segments with the highest *f*_2_-statistics between African cattle and the Muturu + Sahiwal model. We took 3 different size bins for nonoverlapping windows (1, 10, and 50 kb) and chose 4 African cattle breeds with sufficient sample size for the analyses: Butana (East, *n* = 20), Kenana (East, *n* = 13), Baoule (West, *n* = 7), and N’Dama Gambia (West, *n* = 13). Subsequently, we calculated *f*_4_-statistics of the form *f*_4_(African buffalo, X; Muturu + Sahiwal, African cattle) for a chosen set of 9 outgroups (indicines and noncattle Bovini species) using the top 1% genomic segments as well as the whole genome. The horizontal bars represent ±3 standard error measures estimated by 5-cM block jackknifing.

To distinguish different scenarios explaining the Southeast Asian indicine affinity of the African cattle breeds, we investigated genomic segments enriched for the affinity signal. For this, we took top 1% genomic bins for *f2*-statistics between African cattle and the model (Muturu + Sahiwal) after splitting genomes into 1-, 10-, and 50-kb nonoverlapping bins and calculated *f*_4_-statistics of the form *f*_4_(African buffalo, X; Muturu + Sahiwal, African cattle) using the top 1% bins only. We expect that the top 1% bins are enriched for the genomic segments inherited from the third source related to Southeast Asian indicine. Compared with the genome-wide *f*_4_-statistics using all bins, top 1% bins show more positive *f*_4_-statistics when the bins are small (1 or 10 kb) and population X is Jian (Fig. 4; supplementary table S9, Supplementary Material online). When population X is bantengs or gayals that are known to have admixed with Chinese cattle breeds (Chen et al. 2018; Wu et al. 2018), *f*_4_-statistics still show more positive signals with the top 1% bins but to a lesser degree than when Jian is used.

Taken together, these results favor a scenario that African cattle have a single indicine source that is closely related to North Indian indicines but is ancestrally related to Southeast Asian indicines to a small degree. Such a source population is expected to have short genomic segments shared with Southeast Asian indicines distributed across the genome. An independent gene flow from a Southeast Asian indicine source is expected to result in longer genomic segments of the Southeast Asian indicine origin, thus does not explain a lack of signal with 50 kb binning. Likewise, a shared introgression from an outgroup (e.g. banteng or gayal) to Southeast Asian indicines and African cattle would have produced a stronger enrichment signal with these outgroups than with Southeast Asian indicines themselves.

Finally, we tested if South Indian breeds, which are geographically and culturally closer to Southeast Asia than North India, match to the genetic profile of the indicine source of African cattle. Using 2 individuals belonging to Vechur and Kangayam breeds as a source replacing Sahiwal in qpAdm modeling, we find that all 27 breeds are adequately modeled as Muturu + SouthIndian with the base reference set (*P* > 0.05; supplementary table S10, Supplementary Material online). Still, when adding Jian to the reference set, we find that only 5 out of 27 breeds are adequately modeled, and they all have only a small amount of indicine ancestry (Baoule, Kapsiki, N’Dama Gambia, N’Dama Senegal, and N’Dama). This suggests that the currently available South Indian indicine genomes cannot explain the Southeast Asian indicine affinity in African cattle. Therefore, the true indicine source remains elusive.

## Discussion

In this study, we inferred the source of admixture that occurred in African cattle using large-scale sequencing data of present-day and ancient bovids. The multiple evidences in our analyses explicitly support that the secondary admixture from the basal taurine lineage significantly contributed to the formation of African taurine populations (ca. 10% to 20%). This finding is in agreement with the distinct position of African taurine on present-day cattle populations. In the previous studies suggesting the admixture as a major source of African taurine uniqueness, the local aurochs population in North Africa has been suggested as a plausible source of admixture (Decker et al. 2014; Verdugo et al. 2019). We also observe that Moroccan aurochs show higher affinity with the African taurine breeds than with Eurasian taurines (supplementary figs. S2 and S3, Supplementary Material online). Given the geographical proximity of Moroccan aurochs to the geographic origin of African taurine population (Verdugo et al. 2019), we believe that North African aurochs introgression presumably contributed to the divergence of African taurine from Eurasian taurine.

It is critically important to increase the inventory of ancient genomes of African cattle and aurochs to fully resolve the geographic and temporal details of the basal taurine admixture in African taurine populations. A very recent study of Iron Age cattle from the North-Eastern Maghreb indeed provided new information for the origins of African taurine (Ginja et al. 2023). We also note that African aurochs introgression is unlikely to explain all of the admixture signals (∼20%) from the basal taurine lineage. For example, a previous study reported the genetic affinity between the Moroccan aurochs and the Bronze Age southern Levantine cattle (Verdugo et al. 2019). Because southern Levant is considered as a source region for the early African taurine cattle, it is possible that a substantial fraction of the aurochs introgression signal in African taurine may have been inherited from their Levantine cattle ancestors, thus likely coming from Levantine aurochs. Due to the extremely low coverage of the Levantine cattle genomes available, we could not robustly test relevant hypotheses such as (i) if Bronze Age Levantine cattle are a better source for African taurine than Iran_BronzeAge and (ii) if African taurine has a higher amount of aurochs introgression than the Levantine cattle had (supplementary table S11, Supplementary Material online).

In our African admixed breeds, we found that their indicine ancestry has genetic affinity to Southeast Asian indicines in addition to what the North Indian indicines alone can explain. This pattern may be explained by 2 distinct hypotheses: (i) the true single indicine source of African cattle had a genetic profile intermediate of the North Indian and Southeast Asian indicines, or (ii) there were 2 or more streams of indicine gene flow into African cattle among which 1 source was related to the Southeast Asian indicines. We suggest that the former hypothesis may provide a more likely scenario given the currently available evidence. First, the Southeast Asian indicine affinity of African cattle is enriched in the genomic segments showing a high divergence between African cattle and the admixture model of Muturu + Sahiwal (Fig. 4). Second, this enrichment is driven by short genomic segments, which is unlikely under the scenario of a direct admixture with a Southeast Asian indicine source in a couple of hundred generations (Fig. 4).

Interestingly, we observe a clear difference between the indicine ancestry patterns of West and East African breeds (supplementary fig. S9, Supplementary Material online). The indicine ancestry proportion of West African breeds is much more variable than those of East African breeds. Based on our admixture dating, we infer that the recent and sporadic admixture events that might be involved in multiple source populations contributed to the variable admixture proportion in West Africa (supplementary fig. S10 and tables S12 and S13, Supplementary Material online).

The subtle genetic difference between the North Indian indicines and the indicine ancestry of African cattle calls for a fine-resolution study of the indicine genetic diversity. The indicine cattle population is believed to have spread from North India across South Asia between 5,500 and 4,000 yBP and entered East Asia between 3,500 and 2,500 yBP (Chen et al. 2010; Utsunomiya et al. 2019). We hypothesize that the yet-to-be-sampled true ancestral populations of the African indicine ancestry may have been formed during this historical process by incorporating genetic elements from local populations related to Southeast Asian indicines. Indeed, 2 distinct mitochondrial haplogroups have been reported within the Indian subcontinent (Chen et al. 2010), of which the genetic diversity of mitochondrial haplogroup I2 might have been contributed by local wild cattle populations other than North Indian ones. South Indian cattle breeds in our data set do not match to the expected genetic profile of the true indicine source, but there are only 2 individuals from 2 different breeds (supplementary table S10, Supplementary Material online). It may need more diverse breeds to fully represent genetic diversity across the Indian subcontinent including ancient cattle genomes, to enable us to identify the true indicine source(s) in African cattle.

In conclusion, the results presented here illuminate the genetic makeup of African cattle in a fine resolution and provide new hypotheses to be tested. The strong support for aurochs introgression into the earliest cattle in Africa is far-reaching in the context of understanding the rapid success of cattle adaptation to new environmental challenges (e.g. climate and diseases). In addition, the detailed genetic profile of the indicine ancestry of African cattle will give a new insight into understanding the historical relationship between African and Asian civilizations.

## Materials and Methods

### Modern Genome Data Processing

All modern genome data were uniformly processed following our previous study (Kim et al. 2020) (supplementary table S1, Supplementary Material online). We first examined a per-base sequence quality for the raw sequence reads using the fastQC software v0.11.8 (https://www.bioinformatics.babraham.ac.uk/projects/fastqc/) and removed low-quality bases and artifact sequences using Trimmomatic v0.39 (Bolger et al. 2014). The high-quality sequence reads were mapped against the bovine reference genome (ARS-UCD1.2) using bwa mem v0.7.17 (Li and Durbin 2009) with default parameters. We then used SAMtools v1.9 (Li et al. 2009) to sort BAM files and create index files. For the mapped reads, potential PCR duplicates were identified using the “MarkDuplicates” of Picard v2.20.2 (http://broadinstitute.github.io/picard). The “BaseRecalibrator” and “PrintReads” of GATK Genome Analysis Toolkit v3.8 (GATK) (McKenna et al. 2010) were used to perform base quality score recalibration (BQSR). The known variants file (ARS1.2PlusY_BQSR_v3.vcf.gz) provided by the 1000 Bull Genomes Project (http://www.1000bullgenomes.com/) was used for masking known sites for all individuals except the African buffalo, goat, and water buffalo. The before/after BQSR reports were checked by running “AnalyzeCovariates” to ensure that base quality scores are corrected as expected. For the 3 outgroup species, African buffalo, goat, and water buffalo that are overcorrected by the known variants file, we performed an initial round of variant calling on unrecalibrated data. We then performed BQSR by feeding the variants obtained from the initial variant calling, as known sites to BaseRecalibrator and finally checked the convergence of base quality improvement.

For the calling of the candidate SNPs from the BAM files, we created a GVCF file using “HaplotypeCaller” in GATK with “-ERC GVCF” option. Individual GVCF files were merged by breeds using “CombineGVCFs” in GATK. We called and selected candidate SNPs from these combined GVCF files using “GenotypeGVCFs” and “SelectVariants,” respectively. To avoid possible false-positive calls, we filtered out low-quality variants using “VariantFiltration” of GATK with the following criteria: (i) SNPs with mean DP (for all individuals) of <1/5x or >5x (x: overall mean sequencing depth across all SNP sites), (ii) quality by depth of <2, (iii) phred-scaled variant quality score (QUAL) of <30, (iv) strand odds ratio of >3, (v) Fisher strand of >60, (vi) mapping quality of <40, (vii) mapping quality rank sum test of ≤12.5, (viii) read pos rank sum test of ≤8, and (ix) ExcessHet of >10 were filtered. We then excluded nonbiallelic SNPs and genotyped each variant based on its genotype likelihood. Specifically, we took the most likely genotype for each individual and variant if its normalized likelihood exceeded 0.9; otherwise, we treated the genotype as missing data. To account for the genomic differences between cattle, including *B. taurus* and *B. indicus*, and the outgroup, consisting of Bovini species other than cattle, we separately computed the minor allele frequency (MAF) and missingness for each variant in cattle and the outgroup. Next, we filtered out SNPs that met one or more of the following criteria: (i) not located in an autosome, (ii) a transition mutation, (iii) MAF < 0.05 in cattle and <0.15 in the outgroup, (iv) missingness > 0.2 in cattle, or (v) missingness > 0.6 in the outgroup. To exclude spurious genomic regions, we removed SNPs within genomic windows of the bovine reference genome (ARS-UCD1.2) that met one or more of the following criteria: (i) 35-bp windows with low mappability (*c* < 3), following Heng Li's 35mer filter with *r* = 0.5 (https://lh3lh3.users.sourceforge.net/snpable.shtml), (ii) 500-bp windows with high or low GC contents (top 2.5% or low 2.5%), (iii) 500-bp windows with high or low coverage (top 2.5% or low 2.5%), (iv) 500-bp windows with average repeat contents per base > 0.9, or (v) 500-bp windows harboring one or more noncalled positions in the reference (*N*/*n*). Subsequently, we eliminated SNPs that were less than 52 bp away from the next SNP on the right. Finally, we identified nuclear mitochondrial DNA segments (NUMT) regions in the bovine reference genome by aligning the mitochondrial sequence with the autosome sequence using BLASTN version 2.11.0+ and discarded SNPs located in those regions (Altschul et al. 1997). The resulting set of 5,581,829 autosomal transversion SNPs was utilized for all subsequent analyses.

### Ancient Genome Data Processing

Raw read data of all published ancient samples except the British aurochs (CPC98) were processed with EAGER v.1.92.55 (Peltzer et al. 2016), a pipeline for processing ancient DNA sequences. Specifically, Illumina adapter sequences were trimmed using AdapterRemoval 2.3.1 (Schubert et al. 2016). For paired-end sequencing data of ancient Shimao cattle individuals, overlapping read pairs were merged into single read using AdapterRemoval 2.3.1 (Schubert et al. 2016). The trimmed reads were then mapped to the bovine reference genome (ARS-UCD1.2) using bwa aln/samse v.0.7.17 (Li and Durbin 2009) with the relaxed edit distance parameter (-n 0.01). For non-uracil DNA glycosylase (UDG) libraries, seeding was disabled by adding an additional parameter (-l 9999). Duplicates were then removed using DeDup v.0.12.8 (Peltzer et al. 2016) and reads with low mapping quality (Phred-scaled mapping quality score < 30) were filtered out using SAMtools v.1.9 (Li et al. 2009). To reduce the effect of postmortem damage on subsequent analysis, we trimmed BAM files of ancient samples using the trimBam function on BamUtils v.0.15 (Jun et al. 2015) by soft-masking up to 10 bp on both ends of each read, depending on whether the library was treated with UDG or not. The UDG treatment was speculated on the basis of DNA misincorporation pattern per library using mapDamage v.2.2.1 (Jónsson et al. 2013). To call pseudo-haploid genotypes, we randomly sampled 1 high-quality base (Phred-scaled base quality score > 30) using pileupCaller (https://github.com/stschiff/sequenceTools) for each of the 5.58 million transversion SNPs discovered from the modern genome data processing. We used trimmed BAM files for transition SNPs and untrimmed BAM files for transversion SNPs.

The British aurochs data (Park et al. 2015) were processed following the procedure in Verdugo et al. (2019). We first examined a per-base sequence quality for the raw sequence reads using the fastQC software v0.11.8 (https://www.bioinformatics.babraham.ac.uk/projects/fastqc/). Adapters were removed using Cutadapt v2.7 (Martin 2011) with a minimum overlap length of 1 bp between the read and the adapter sequence and a minimum read length of 30 bp. The clean reads were then aligned to the ARS-UCD1.2 reference genome using bwa aln v0.7.17 (Li and Durbin 2009) with seeding disabled (-l 1024) and the bwa samse with the option -r for defining read groups. SAMtools v1.9 (Li et al. 2009) was used to convert SAM files to BAM files, sort BAM files, and remove PCR duplicates (rmdup -s). The BAM files were filtered using SAMtools v1.9 (Li et al. 2009) for removing unaligned reads (view -F4). To reduce the effect of postmortem damages in genotyping, we trimmed 8 bp from both ends of the reads using BamUtils v1.0.14 (Jun et al. 2015). We then created a pileup using SAMtools mpileup v1.9 (Li et al. 2009) with -R and -B flags and call “pseudo-haploid” genotypes by randomly drawing a single high-quality base (base quality score of 30 or higher) from the high-quality mapped reads (mapping quality score of 30 or higher) (-q30 and -Q30) using the pileupCaller program implemented in the sequenceTools v1.4.0 (https://github.com/stschiff/sequenceTools).

### Principal Component Analysis

For principal component analysis (PCA), we first calculated eigenvectors with 543 present-day cattle genomes and projected 38 ancient genomes on the calculated eigenvectors using smartpca v.18140 (Patterson et al. 2006) with the option “lsqproject: YES.” For an alternative visual summary of the relationship of African cattle with the other cattle populations, we calculated eigenvectors with 394 present-day cattle genomes excluding Chinese cattle individuals (North Central China, North West China, and South China).

### Outgroup *f*_3_- and *f*_4_-Statistics

We used the *f*_4_-statistics of the form *f*_4_(African buffalo, Sahiwal; African cattle, representative taurine breeds) to quantify the degree of indicine admixture in African cattle with ADMIXTOOLS v7.0.2 (Patterson et al. 2012). We chose the following 5 as representative taurine breeds (4 ancient and 1 present-day): Anatolia_Neolithic, Balkans_Neolithic, Iran_BronzeAge, Shimao_High, and Simmental. We used Sahiwal and African buffalo to represent a South Asian indicine and an outgroup to both taurine and indicine cattle, respectively.

To measure the genetic affinity between taurine breeds and ancient aurochs from different regions (Anatolian, Armenian, British, and Moroccan aurochs), we computed outgroup *f*_3_ of the form *f*_3_(African buffalo; aurochs, taurine) and *f*_4_-statistics of the form *f*_4_(African buffalo, aurochs; Anatolia_Neolithic, taurine). As taurine breeds, we used breeds from the following regions: ancients (Anatolia_Neolithic, Balkans_Neolithic, Iran_BronzeAge, and Shimao_High), Europe, Northwest China, Northeast Asia, and Africa (N’Dama and Muturu).

### Admixture Graph Construction with qpGraph

We employed the “qpgraph” function from the ADMIXTOOLS2 package (Maier et al. 2023) to identify the most plausible admixture graph topologies. Initially, we constructed the best-fitted backbone graph (topology A-1) with the highest likelihood score and the smallest worst *z*-score for 6 populations: African buffalo, Sahiwal, Armenian aurochs, British aurochs, Moroccan aurochs, and Iran_BronzeAge. This backbone graph involved 2 gene flow events. We then added Muturu onto the backbone graph and explored all potential admixture graphs with and without a gene flow into Muturu. To identify the optimal topology without gene flow into Muturu, we selected the one (topology B-1) with the highest likelihood score and the least worst *z*-score, where the *z*-scores are calculated by 5-cM block jackknifing. Similarly, we chose 2 best topologies (topologies B-2 and B-3) involving a gene flow into Muturu, where their likelihood scores were nearly indistinguishable and their worst *z*-scores were less than 3. To assess the robustness of our choice regarding ancient taurine, we repeated our analysis after substituting Iran_BronzeAge with Anatolia_Neolithic in the topologies B-1, B-2, and B-3. Finally, we added Jian onto the topology B-2, which contains a direct gene flow from Moroccan aurochs into Muturu, and explored all potential admixture graphs with and without a gene flow into Jian. We selected the best-fitted admixture graph with and without a gene flow into Jian, respectively.

To compare the model fit between 2 competing models, we employed the “qpgraph_resample_multi” function from the ADMIXTOOLS2 package (Maier et al. 2023) to estimate bootstrap-resampled graph fits. The procedure involved the following steps. First, we split the genome into nonoverlapping 5-cM intervals. Second, we performed bootstrap resampling of the 5-cM intervals by sampling as many intervals as the total number of intervals with replacement. Third, we fitted each of the 2 models under comparison with resampled data and estimated branch lengths and admixture proportions. Fourth, using genomic intervals not included in bootstrap resampling, we computed likelihood scores for each model. This resampling approach enables us to prevent overfitting and facilitates a reliable comparison between models with varying complexities. To ensure robustness, we repeated the estimation of bootstrap-resampled graph fits 1,000 times. Subsequently, we derived an empirical *P*-value by calculating the proportion of bootstrap replicates where the likelihood score of model B-1 exceeded that of models B-2 or B-3 and dividing this by the total number of replicates (1,000). We preferred the use of the empirical *P*-value over the *P*-value obtained from paired *t*-tests, as paired *t*-tests assume that the score difference follows a normal distribution.

### Robustness Test of qpGraph Results via Simulation

To test the robustness of our qpGraph analysis, we simulated genome sequence data according to the best topology without (B-1) and with a gene flow into Muturu (B-2 and B-3) using msprime 1.2.0 (Baumdicker et al. 2022). We used a mutation rate of 1.25 × 10^−8^ per base pair per generation, a recombination rate of 1.60 × 10^−8^ per base pair per generation, an effective population size of 10,000 for all populations, and generation time of 6 yr. Each simulation run consisted of 800 unlinked 50-kb regions, resulting in ca. 200,000 SNPs, matching the number of SNPs used in the qpGraph analysis. To simulate genotype data resembling the effective sample size of each population considering missingness, we decided the number of haploid individuals for each population by taking the average number of chromosomes present in each population across SNPs, taking into account that pseudo-haploid genotype calls of ancient individuals in fact represent 1 chromosome per individual. The populations and corresponding sample sizes were as follows: African buffalo (*n* = 2, diploid), Sahiwal (*n* = 2, diploid), Armenian aurochs (*n* = 1, haploid), British aurochs (*n* = 1, haploid), Moroccan aurochs (*n* = 1, haploid), Iran_BronzeAge (*n* = 2, haploid), and Muturu (*n* = 9, diploid). We simulated 100 independent genotype data sets for each topology.

Given the lack of an explicit demographic model for cattle, we adopted heuristic split times between branches and admixture proportions based on estimates derived from our qpGraph analysis. To test the impact of the split time between African buffalo and cattle on the analysis, we used 2 distinct split times: 300,000 and 3,000,000 yBP. The split times used are as follows:

- African buffalo—cattle: 300,000 or 3,000,000 yBP
- Indicine—all taurine: 180,000 yBP
- Deep taurine branch—other taurine: 60,000 yBP
- British aurochs—remained taurine: 36,000 yBP
- Armenian aurochs—remained taurine: 24,000 yBP
- Moroccan aurochs—remained taurine: 15,000 yBP
- Introgressed taurine into Sahiwal—Iran_BronzeAge: 9,000 yBP
- Muturu—Iran_BronzeAge: 6,000 yBP
- Introgressed aurochs into Muturu—Moroccan aurochs: 3,600 yBP (B-2)
- Introgressed aurochs into Muturu—Introgressed aurochs into Moroccan aurochs: 30,000 yBP (B-3)

Admixture proportions and times are as follows:

- Taurine introgression into Sahiwal: 6,000 yBP, 20%
- Deep taurine introgression into Moroccan aurochs: 12,000 yBP, 40%
- Moroccan aurochs introgression into Muturu: 3,000 yBP, 40% (B-2)
- Deep taurine introgression into Muturu: 3,000 yBP, 20% (B-3)

For each simulated genotype data, we conducted a comparative assessment of the fits between topology B-1 and B-2 or B-3, employing the same model comparison procedure as in the qpGraph analysis. For the data sets simulated without gene flow (B-1), we determined the number of simulated data sets where graphs with gene flow (B-2 or B-3) exhibited a significantly better fit (empirical *P* < 0.05) compared with the true graph B-1. This measurement served as an indicator of false positives for the introgression signal. On the other hand, for the data sets simulated with gene flow (B-2 and B-3), we counted the number of simulated data sets where graphs without gene flow (B-1) adequately explained the data without a significant reduction in model fit (empirical *P* ≥ 0.05). This measurement provided us with insights into false negatives, i.e. scenarios where the presence of gene flow may not be accurately detected. By employing this comparative approach, we aimed to gauge the performance and accuracy of our model in identifying introgression events and distinguishing between data sets with and without gene flow.

### TreeMix Analysis

We inferred a population-level phylogeny and migration edges using the maximum likelihood approach implemented in TreeMix (Pickrell and Pritchard 2012). We used the same genotype data and same 7 populations as the qpGraph analysis to independently support qpGraph-based results (see “Admixture Graph Construction with qpGraph”). We inferred the maximum likelihood population graph with 0 to 3 migration edges allowed. We used 200 SNPs per block for estimating the covariance matrix (“-k 200”). We set African buffalo as an outgroup (“-root AfricanBuffalo”) and performed a round of global rearrangements after adding all populations into the graph with the “-global” option. We used the flag -noss to avoid overcorrection for sample size as we have a population with a single individual.

### Admixture Modeling of Muturu with qpAdm

We modeled Muturu, an African taurine breed without an indicine admixture signal, using the qpAdm v.1520 implemented in ADMIXTOOLS v7.0.2 (Patterson et al. 2012). First, we modeled Muturu as a mixture of Iran_BronzeAge and the Moroccan aurochs, corresponding to the topology B-2 (Fig. 2A), with the following 5 populations as the references: African buffalo (*n* = 2), Sahiwal (*n* = 2), Balkans_Neolithic (*n* = 16), British aurochs (*n* = 1), and Shimao_High (*n* = 2). Second, we estimated the proportion of a gene flow from the basal taurine lineage to Muturu by using Iran_BronzeAge and one of indicine/outgroup populations as source populations with a set of 3 reference populations: Balkan_Neolithic, British aurochs, and Shimao_High. By restricting the reference populations to the 3 populations closer to Iran_BronzeAge than indicine and symmetrically related to Iran_BronzeAge and Iran_BronzeAge-related ancestry in Muturu, the model becomes blind to the difference between sources (e.g. indicine and basal taurine lineage in the model) branching at deeper positions in the population graph than all reference populations (Skoglund et al. 2017; van de Loosdrecht et al. 2018). This allows us to estimate a proportion of a gene flow from the basal taurine lineage without a sample on that lineage.

### Admixture Modeling of Admixed African Cattle Breeds with qpAdm

We performed 3 sets of qpAdm-based admixture modeling of admixed African cattle breeds to characterize their indicine source in fine resolution (Models I, II, and III). For each set, we modeled admixed African cattle breeds with 2 sources, Muturu and an indicine (North Indian indicine breed Sahiwal for Models I and III, Southeast Asian indicine breed Jian for Model II). For Models I and II, we used the following base set of reference populations: African buffalo (*n* = 2), British aurochs (*n* = 1), Balkan_Neolithic (*n* = 16), and Shimao_High (*n* = 2). For Model III, we added Jian as an additional reference population. We used Jian as a representative of Southeast Asian indicine breeds based on its sample size (*n* = 4) and indicine ancestry estimate (100%) from a previous study (Chen et al. 2018). To check the representativeness of Jian, we also tested a variant of Model III, where we added other Southeast Asian indicine breeds one by one to the references instead of Jian and repeated the qpAdm analysis.

### Localizing of the Southeast Asian Indicine Affinity in African Cattle Genomes

To further characterize the Southeast Asian indicine affinity of African cattle, we investigated genomic segments of African cattle that show most divergence from the admixture model in Model I (Muturu + Sahiwal). To ensure robustness in our analysis, we selected 2 African cattle breeds from East and West Africa with relatively large sample sizes: Butana (East, *n* = 20), Kenana (East, *n* = 13), Baoule (West, *n* = 7), and N’Dama Gambia (West, *n* = 13). Our analysis proceeded in the following steps. First, we split the African cattle genome into nonoverlapping bins of 1-, 10-, and 50-kb sizes. Second, we calculated per-bin *f2*-statistics between the observed allele frequency of African cattle and the expected allele frequency calculated as Muturu + Sahiwal with the qpAdm-based admixture proportions inferred in Model I as weights. Third, for each bin size, we took the top 1% bins for high *f2*-statistics as the most divergent genomic segments from the admixture model, thus likely breaking the admixture models in Model III. Fourth, we calculated *f*_4_-statistics of the form *f*_4_(African buffalo, X; Muturu + Sahiwal, African cattle) for the top 1% bin SNPs only. For population X, we used Jian, Chinese gayal, Bangladesh gayal, Indian gayal, gaur, banteng, domestic yak, American bison, and European bison. More positive *f*_4_-statistics than the same ones with all genome-wide SNPs suggest that the corresponding population X is related to the unknown ancestry component that makes Model III break.

### Admixture Dating with ALDER and DATES

We used ALDER v1.03 (Loh et al. 2013) for the dating of admixture events of the African cattle using the default parameters with a minimum genetic distance (mindis) of 0.5 cM assuming that gene flow occurred as a single event.

For the African cattle breeds with a single individual, we used DATES v4000 (Narasimhan et al. 2019), which allows estimating the admixture date in a single genome. In the parameter file for running DATES, we used the options binsize, 0.001; maxdis, 1.0; runmode, 1; qbin, 10; and lovalfit, 0.45. In every run of ALDER and DATES, we used Muturu and India-Pakistan–North American–South American breeds (supplementary table S1, Supplementary Material online) as a source of African taurine and indicine, respectively.

### GLOBETROTTER Analysis

We performed GLOBETROTTER analysis (Hellenthal et al. 2014) for elucidating the admixture profiles of 26 African cattle breeds, excluding Muturu and N’Dama due to the lack of indicine admixture signal in them. The GLOBETROTTER software exploits haplotype-sharing patterns of the target and reference populations (supplementary table S1, Supplementary Material online) to investigate the evidence of admixture in the target population, estimate the admixture date, and perform the detailed decomposition of genetic profile in the source using the reference populations. We performed the regional analysis approach as described in Hellenthal et al. (2014), where target populations cannot be copied from each other but from the worldwide modern reference populations including African taurine, following the developer's recommendation. In this study, we used fastGLOBETROTTER (Wangkumhang et al. 2022), which uses more efficient algorithms than GLOBETROTTER while showing similar performance.

We jointly phased the modern cattle genome data, including *B. taurus and B. indicus*, without a phased reference panel, using SHAPEIT2 v2.904 with the default setting (Delaneau et al. 2013). Chromosome painting was then carried out with the following 2 steps. For a subset of chromosomes (1, 2, 7, and 12) of all target and reference populations, we first estimated the parameters *N*e and θ using 10 iterations of the expectation–maximization algorithm with “-in” and “-iM” switches in ChromoPainter v2 (Lawson et al. 2012). For regional analysis, we repeated the parameter estimation using target populations as a recipient and reference populations as a donor. Second, we painted both target and reference haplotypes using reference haplotypes with the parameters averaged over all haplotypes (“-n 1319.47 -M 0.00228336”) to obtain a “chunk length” matrix. We also sampled 10 painting haplotypes for each target population using reference haplotypes with their own parameter averaged over individuals of each target population.

Using the chunk length matrix and painting samples, we performed the GLOBETROTTER analysis for both stages 1 (“prop.ind: 1” and “null.ind: 1”) and 2 (“prop.ind: 1” and “null.ind: 0”) in case of the population with multiple individuals and only stage 2 otherwise. At stage 1, GLOBETROTTER normalizes coancestry curves by use of null individuals to alleviate LD caused by bottleneck after admixture, which guarantees the robust inference of admixture in the target population. We measured the *P*-value of the admixture using 100 bootstrapping replicates with the “prop.ind: 0” and “bootstrap.date.ind: 1” options. The estimated date of admixture between 1 and 400 generations was regarded as evidence of admixture. Lastly, we adopted the stage 1 result as an estimated date rather than stage 2 to avoid the effect by bottleneck after admixture (Hellenthal et al. 2014) except for the breeds with a single sample. To estimate Southeast Asian indicine admixture proportions in each African breed, we summed proportions of the decomposed sources according to the 5 groups defined by breed origins (supplementary table S8, Supplementary Material online).

## Supplementary Material

msad257_Supplementary_Data

## Data Availability

All raw data in this study are derived from public resources described in supplementary table S1, Supplementary Material online.

## Code Availability

No custom code is used in this study.
