# Supplementary material for: Inference of Admixture Origins in Indigenous African Cattle
Source: Mol Biol Evol. 2023 Nov 23;40(12):msad257. doi: 10.1093/molbev/msad257 (PMC10701095; doi:10.1093/molbev/msad257)
Supplement: msad257_Supplementary_Data [file msad257_supplementary_data.zip › AfricanCattle2 SI r2-v04.docx]

**Supplementary Information**

**Inference of admixture origins in indigenous African cattle**

Kwondo Kim, Donghee Kim, Olivier Hanotte, Charles Lee, Heebal Kim*, Choongwon Jeong*

* Correspondence to: [heebal@snu.ac.kr](mailto:heebal@snu.ac.kr) (H.K.) and [cwjeong@snu.ac.kr](mailto:cwjeong@snu.ac.kr) (C.J.)

**This file includes:**

Supplementary Figures S1 to S10


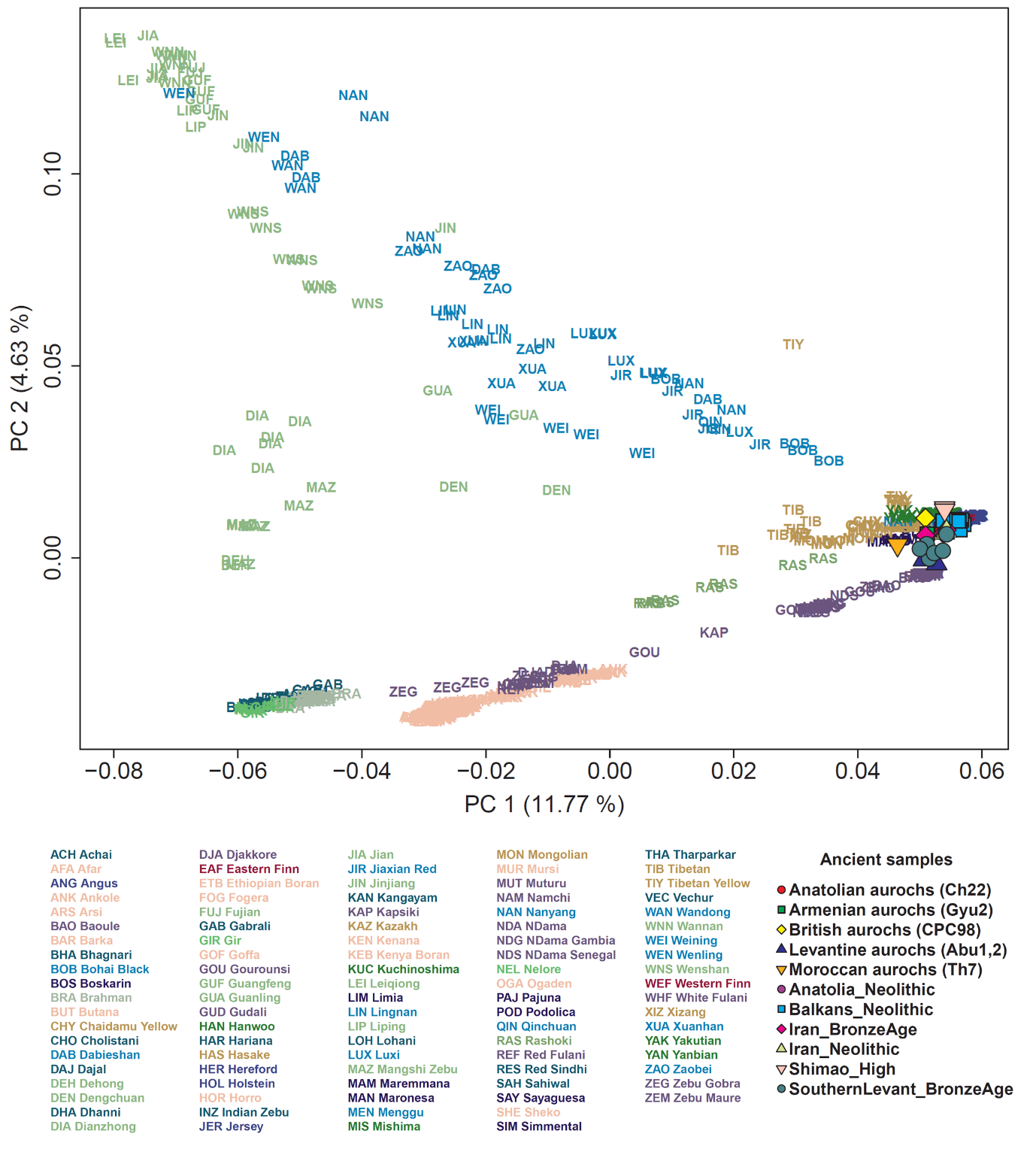


**Supplementary Figure 1.** **The genetic profile of world-wide cattle summarized by the top two PCs calculated from 394 present-day world-wide cattle individuals including both *B. taurus* and *B. indicus*.** Each present-day cattle individual is marked by a three-letter code representing its breed name and colored according to its geographic group. Ancient cattle and aurochs individuals are projected onto the top PCs and marked by color-filled symbols. The proportion of variance explained by each principal component is indicated in the axis labels.


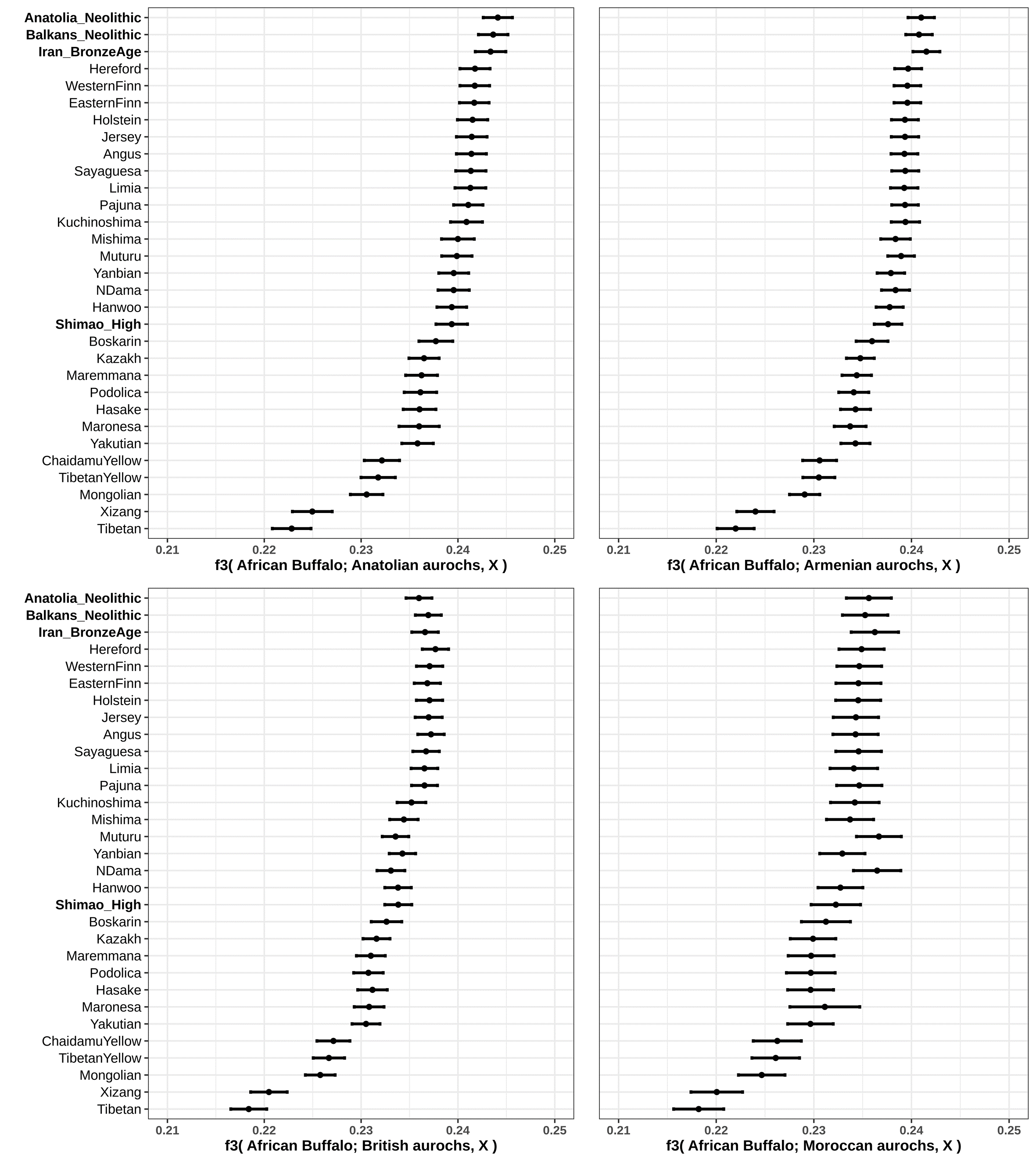


**Supplementary Figure 2. Outgroup *f_3_*-statistics of the form *f3*(African Buffalo; aurochs, taurine).** This is to test the genetic affinity of taurine cattle breeds with four aurochs (Anatolian, Armenian, British, and Moroccan aurochs) using African Buffalo as the outgroup. The ancient genomes are indicated in bold. Circles mark the point estimate of *f_3_*-statistics. Horizontal bars represent ±3 standard error measures (s.e.m.) estimated by 5 cM block jackknifing.


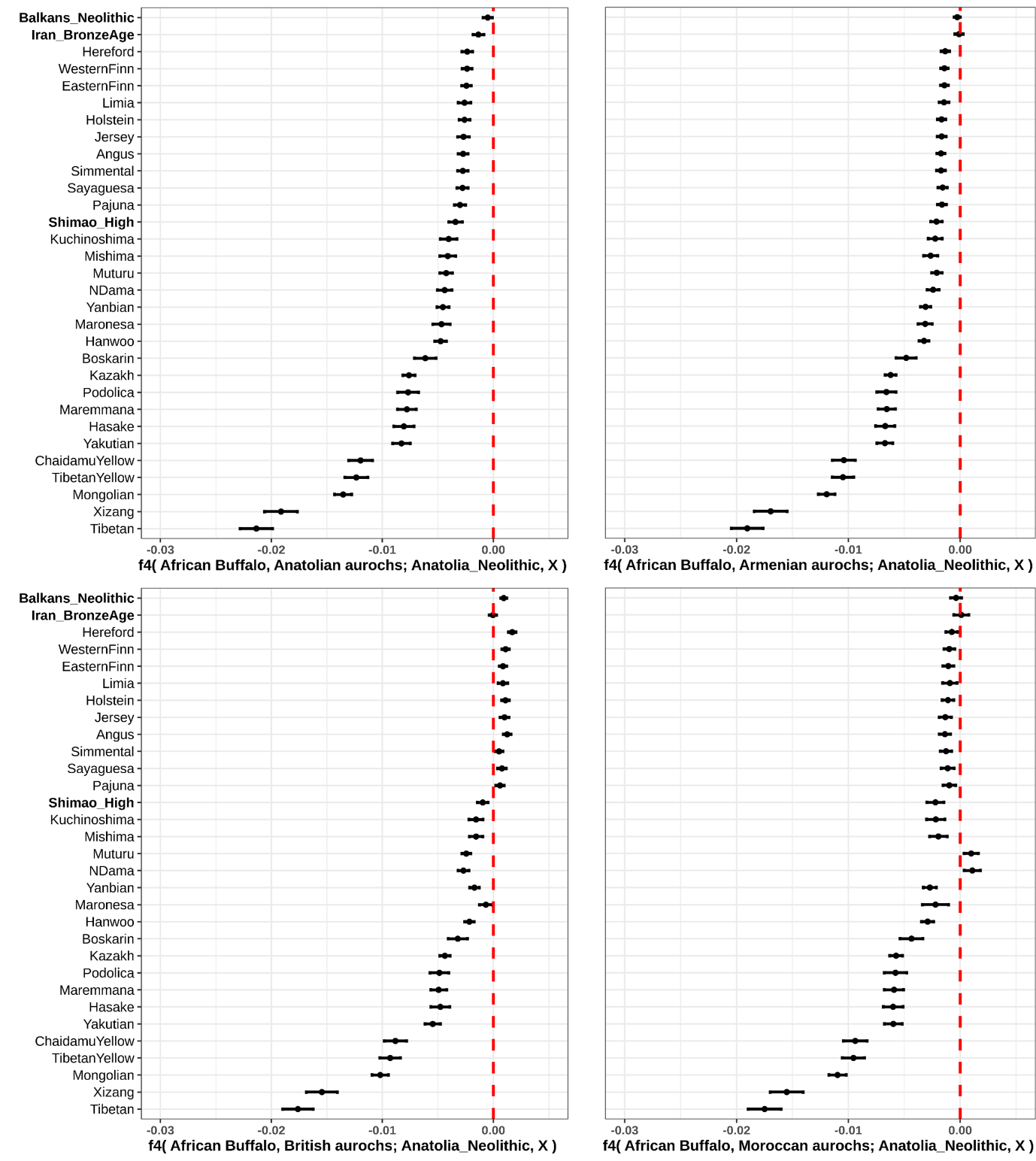


**Supplementary Figure 3**. ***f_4_*-statistics of the form f4(African Buffalo, aurochs; Anatolia_Neolithic, taurine).** This is to measure the genetic affinity of taurine cattle breeds with four aurochs (Anatolian, Armenian, British, and Moroccan aurochs) in comparison to Anatolia_Neolithic. The ancient genomes are indicated in bold. Circles mark the point estimate of *f_4_*-statistics. The red dotted line marks the zero value, implying that the tested taurine breed and Anatolia_Neolithic are equally related to the corresponding aurochs. Horizontal bars represent ±3 s.e.m. estimated by 5 cM block jackknifing.


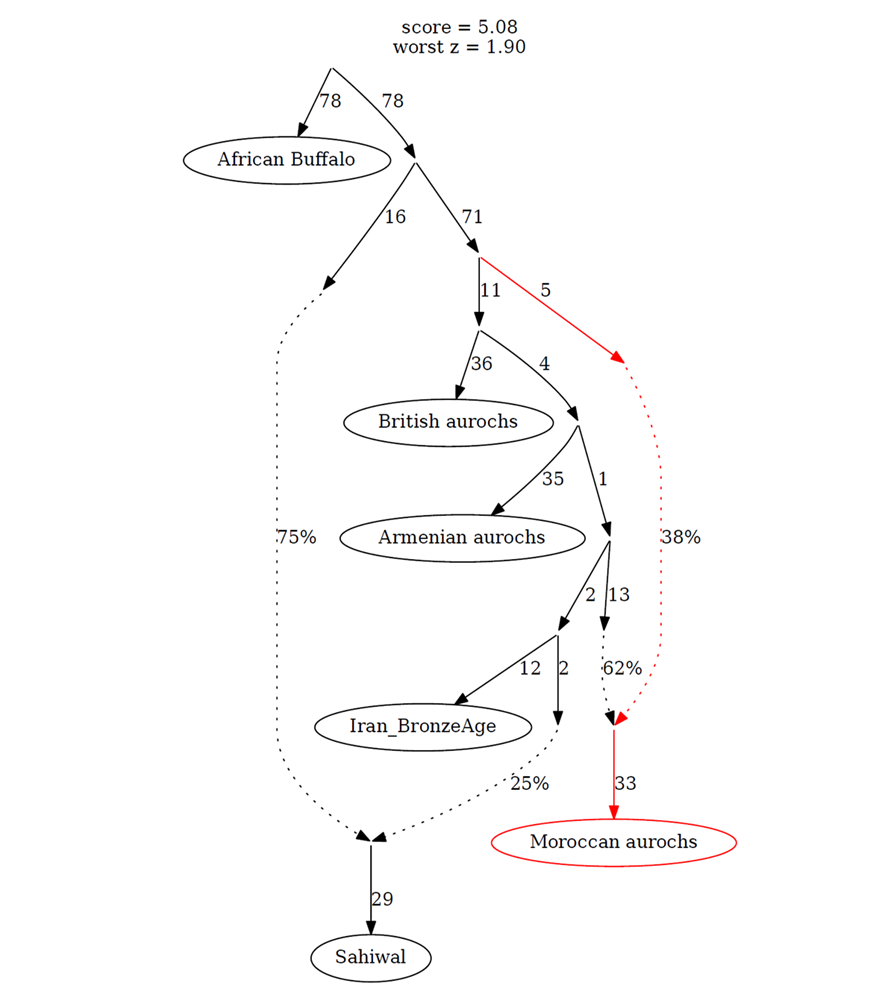


**Supplementary Figure 4. A well-fitted backbone population graph of six populations obtained from qpGraph.** This backbone graph (topology A-1) includes African Buffalo (outgroup), three aurochs (British, Armenian, and Moroccan), a taurine (Iran_BronzeAge) and an indicine cattle (Sahiwal). Edge lengths represent F_st_×1000. This graph includes two gene flow events, and all the observed f-statistics are within 1.9 s.e.m. of the value expected from the graph. The red-colored edges represent a deep taurine contribution into the Moroccan aurochs, with the source split from the other aurochs/taurine populations before the split between British aurochs and others.


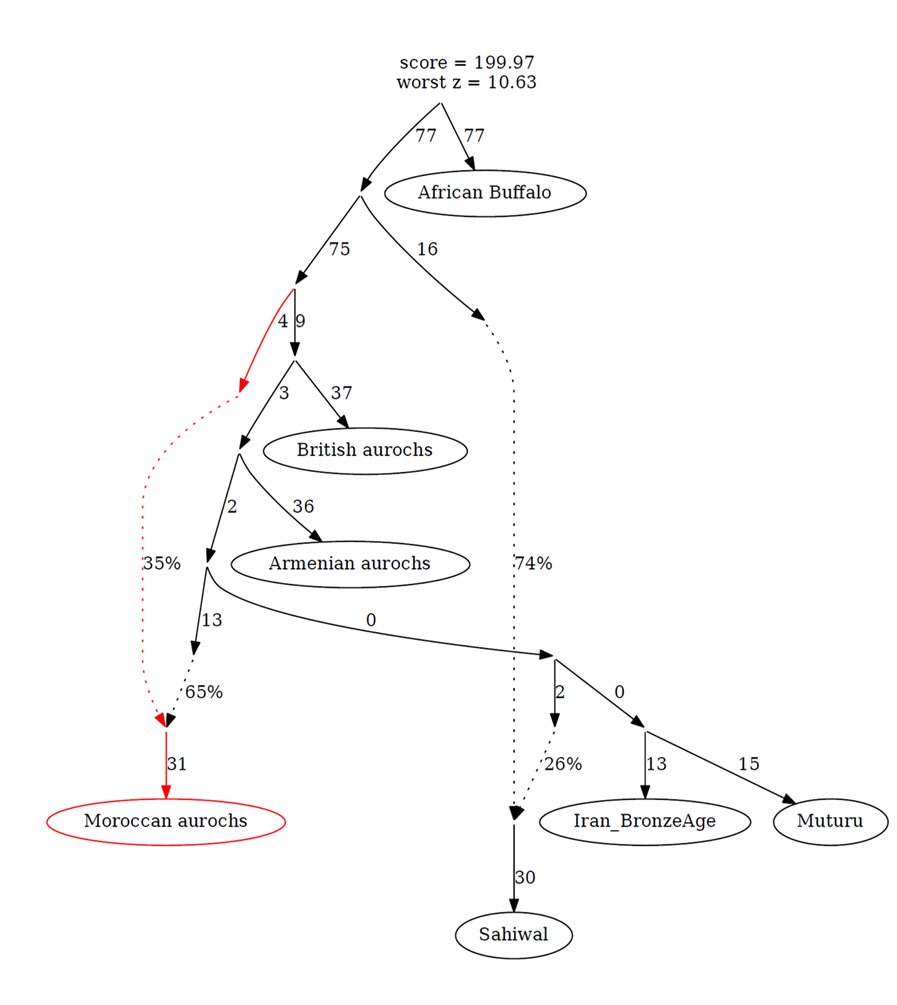


**Supplementary Figure 5**. **The best population graph with Muturu without admixture.** This graph (topology B-1) has the highest score among all possible graphs adding Muturu on top of the backbone graph A-1 without involving gene flow into Muturu. The graph deviates from the observed f-statistics by a large margin, shown by the largest deviation between the observed and expected f-statistics (“worst z”) of the value of 10.63 s.e.m..


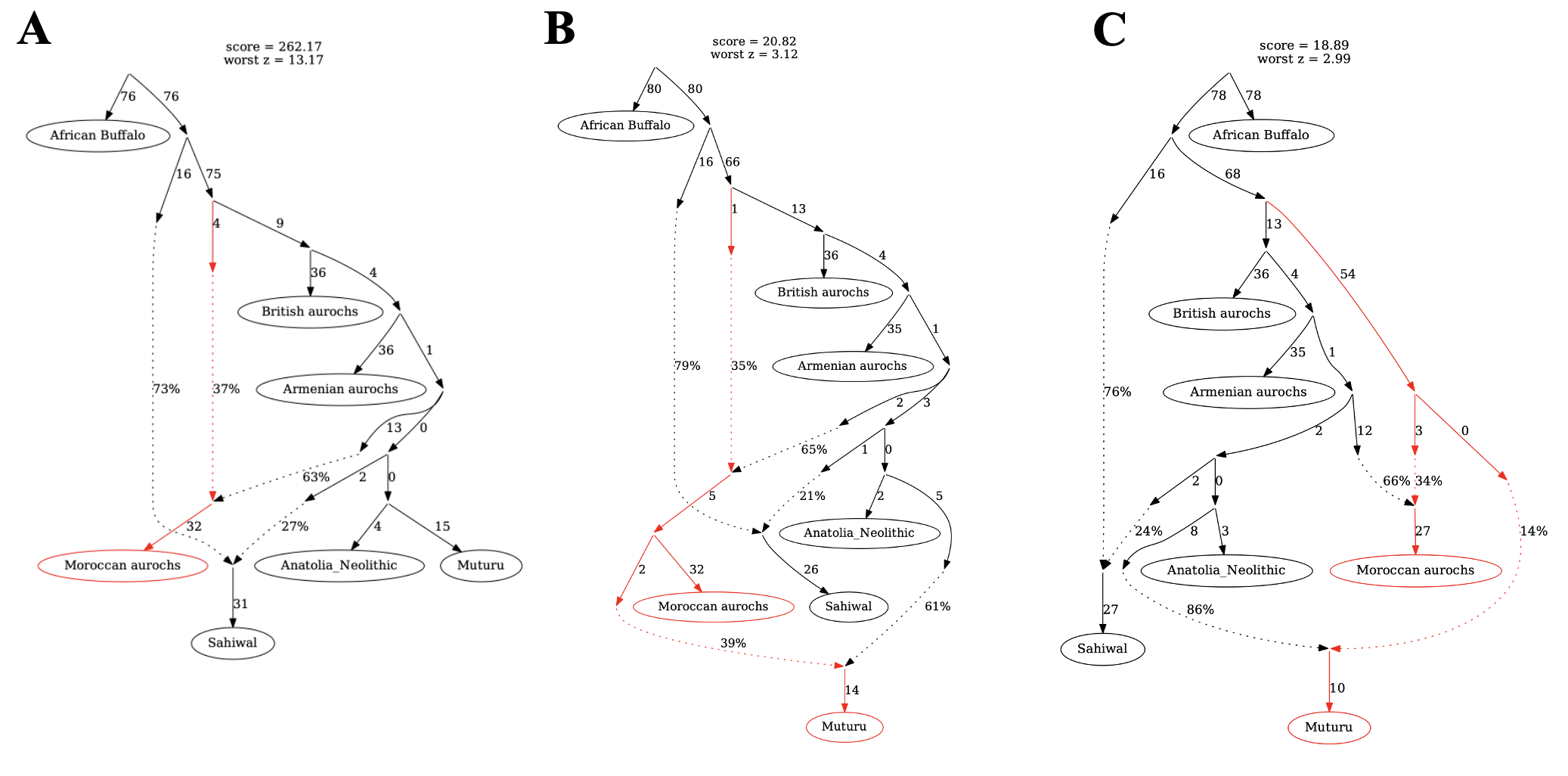


**Supplementary Figure 6**. **The impact of substituting Iran_BronzeAge with Anatolia_Neolithic on qpGraph analysis.** Iran_BronzeAge was substituted with Anatolia_Neolithic in the topology B-1 (A), B-2 (B), and B-3 (C). (A) When we introduced Anatolia_Neolithic into the topology B-1 as a replacement for Iran_BronzeAge, the model consistently failed (worst z score = 13.17). (B, C) Involving a gene flow into Muturu improved the fit considerably (worst z score = 3.12 and 2.99 respectively).


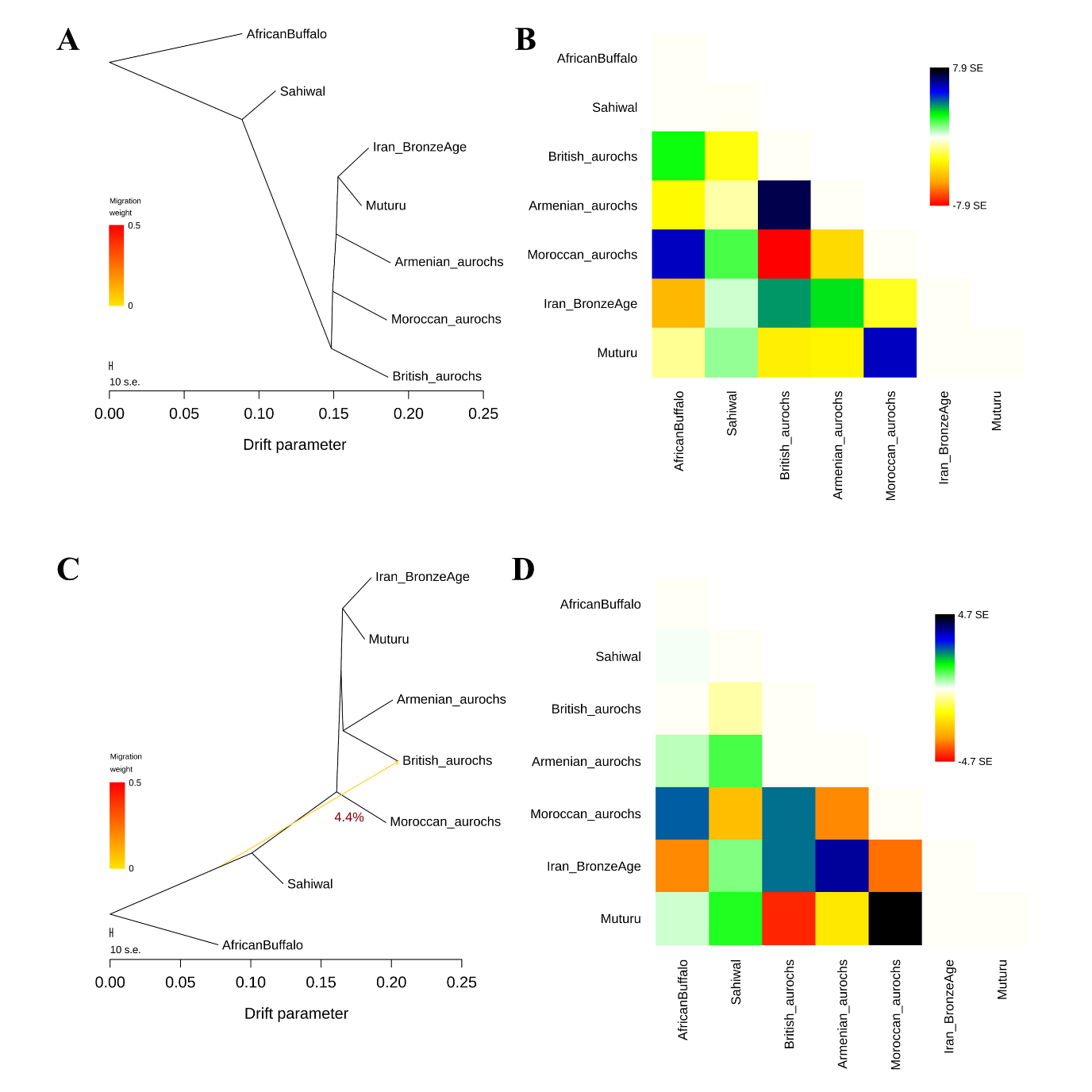


**Supplementary Figure 7**. **The population graphs inferred with TreeMix.** We present (A, C, E, G) the maximum likelihood population graph and (B, D, F, H) its residual allele frequency covariance matrix with the number of migration edges (m) ranging between 0 to 3 (from top to bottom). The scale bars represent ten times the average standard error (s.e.) of the values in the covariance matrix.


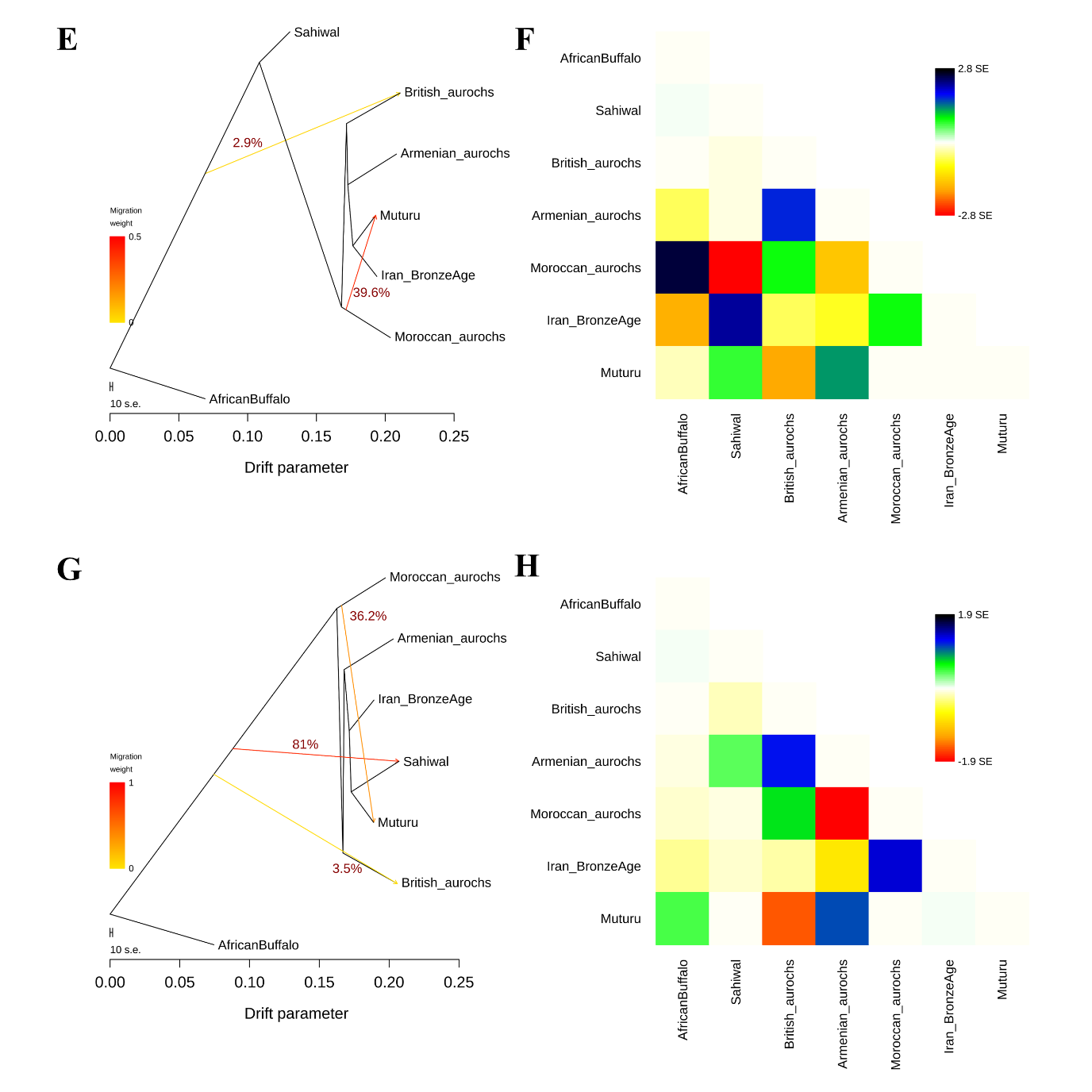


**Supplementary Figure 7**. **The population graphs inferred with TreeMix. (continued)**


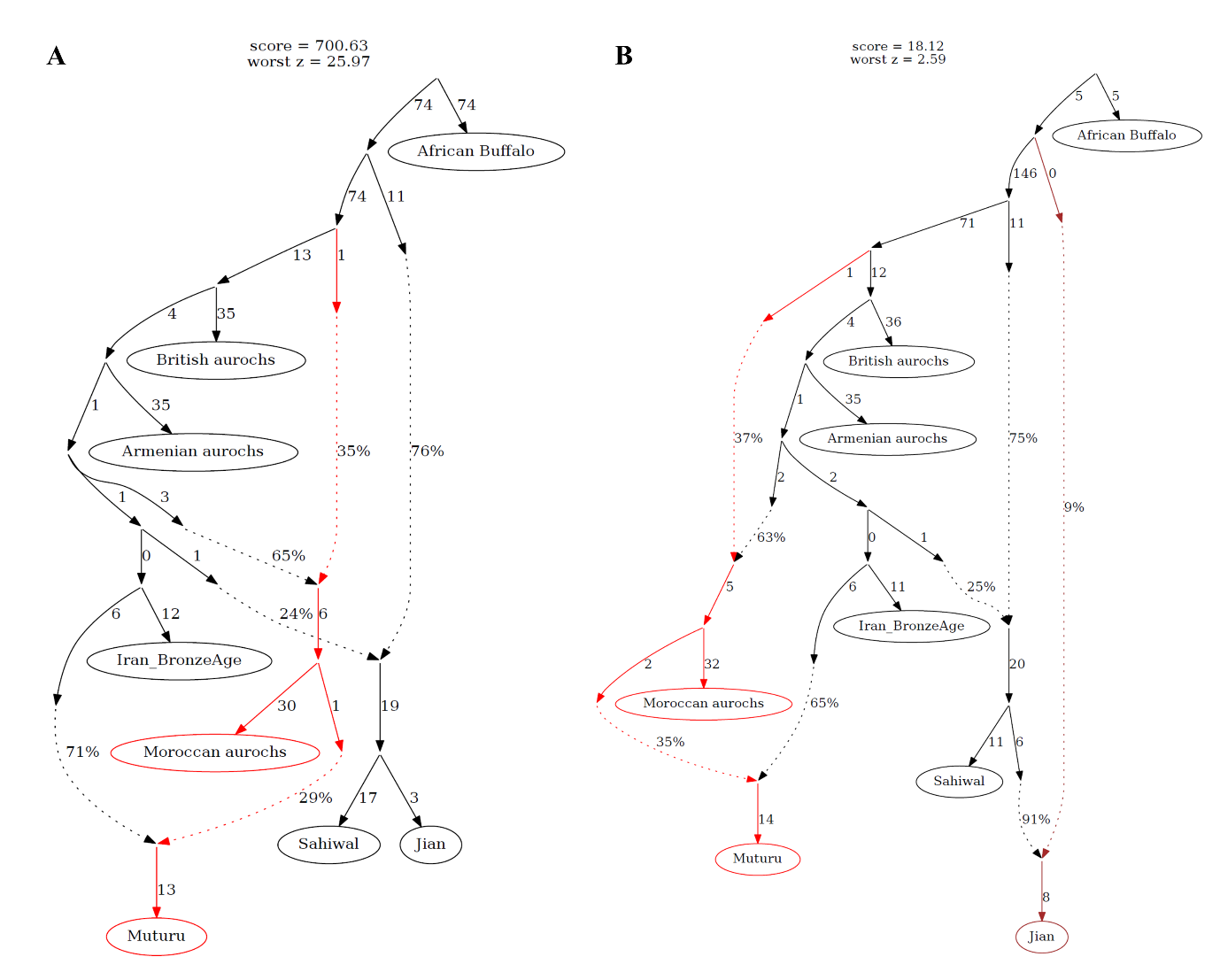


**Supplementary Figure 8**. **The non-cladal relationship of the North Indian and Southeast Asian indicines.** A Southeast Asian indicine breed (Jian) is added to the graph (topology B-2). (A) When Jian is added without admixture, it is modeled as a sister group to Sahiwal, a North Asian indicine. However, the model fails (worst z-score = 25.97). (B) When Jian is modeled as a mixture of a Sahiwal-related branch and a deep branch outgroup to both taurine and indicine (highlighted in brown color), the graph adequately fits the observed data (worst z-score = 2.59).


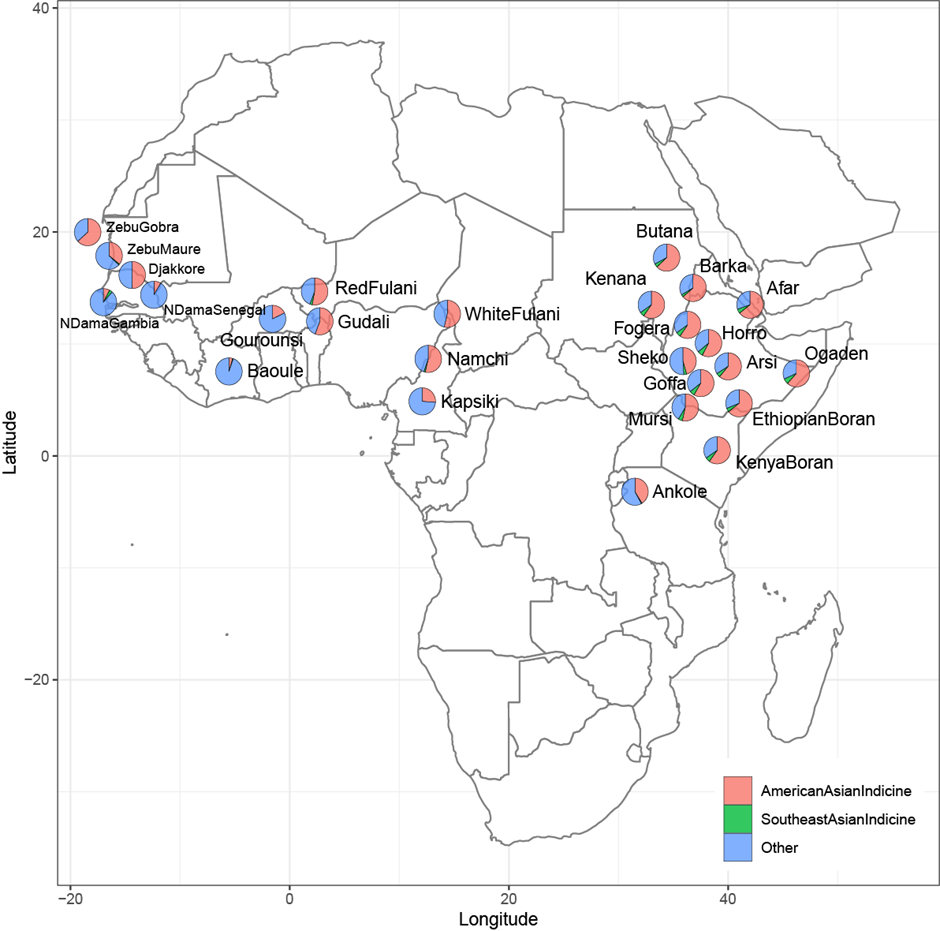


**Supplementary Figure 9. The GLOBETROTTER-based estimates of ancestry proportions of African cattle breeds.** We present the proportion of each breed’s ancestry derived from American/Asian indicine (red), Southeast Asian indicine (green), and others (blue) as a pie chart. The African continent map was generated using “borders” function implemented in ggplot2 package. The geographic location of each breed is retrieved from the following sources: 1) Hanotte et al. 2000 (Molecular ecology. 9(4): 387-396) for Afar, Ankole, Arsi, Ethiopian Boran, Fogera, Horro, Kenya Boran, and Sheko, 2) Hanotte et al. 2002 (Science. 296(5566): 336-339) for Butana and Ogaden, 3) DAGRIS (<http://dagris.ilri.cgiar.org>) for Barka, Goffa, Kenana, and Mursi, and 4) the center of the country of origin as described in Table S1 for Baoule, Djakkore, Gourounsi, Gudali, Kapsiki, Muturu, Namchi, N’Dama Gambia, N’Dama, N’Dama Senegal, Red Fulani, White Fulani, Zebu Gobra, and Zebu Maure.


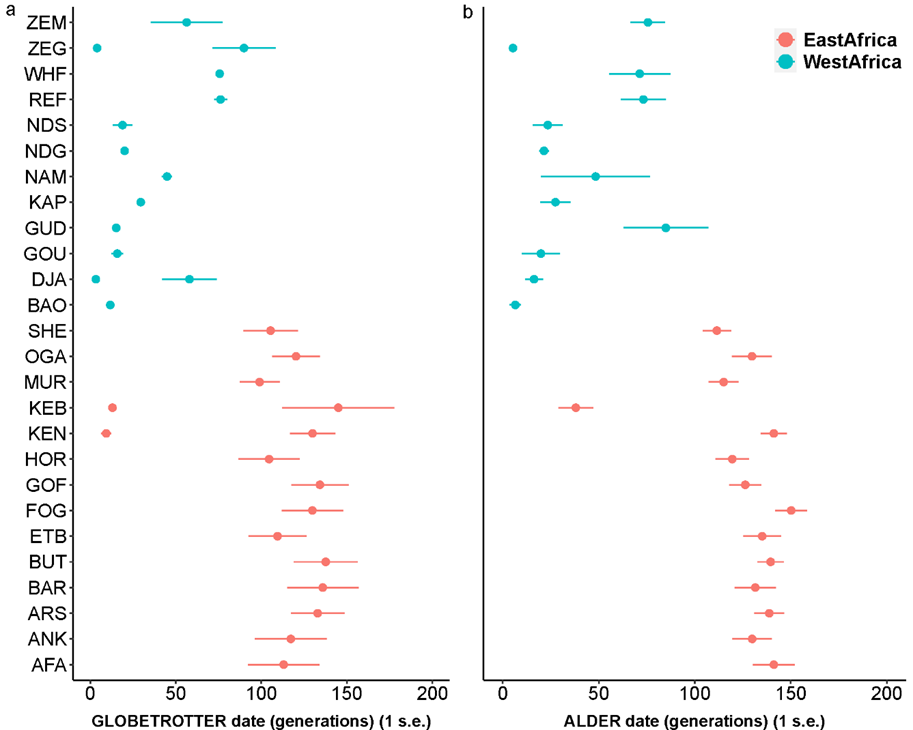


**Supplementary Figure 10.** The inferred dates of admixture events in the African cattle breeds using (a) GLOBETROTTER and (b) ALDER and DATES. Horizontal bars represent ±1 s.e.. The abbreviation of each breed is written in Table S5.
